# Extreme Growth Failure is a Common Presentation of Ligase IV Deficiency

**DOI:** 10.1002/humu.22461

**Published:** 2013-11-08

**Authors:** Jennie E Murray, Louise S Bicknell, Gökhan Yigit, Angela L Duker, Margriet van Kogelenberg, Sara Haghayegh, Dagmar Wieczorek, Hülya Kayserili, Michael H Albert, Carol A Wise, January Brandon, Tjitske Kleefstra, Adilia Warris, Michiel van der Flier, J Steven Bamforth, Kurston Doonanco, Lesley Adès, Alan Ma, Michael Field, Diana Johnson, Fiona Shackley, Helen Firth, C Geoffrey Woods, Peter Nürnberg, Richard A Gatti, Matthew Hurles, Michael B Bober, Bernd Wollnik, Andrew P Jackson

**Affiliations:** 1 MRC Human Genetics Unit, Institute of Genetics and Molecular Medicine, University of EdinburghEdinburgh, UK; 2 Institute of Human Genetics, Centre for Molecular Medicine Cologne (CMMC), University of CologneCologne, Germany; 3 Cologne Excellence Cluster on Cellular Stress Responses in Aging-Associated Diseases (CECAD), University of CologneCologne, Germany; 4 Division of Genetics, Department of Pediatrics, A.I. DuPont Hospital for ChildrenWilmington, Delaware; 5 Wellcome Trust Sanger InstituteCambridge, UK; 6 UCLA Department of Pathology & Laboratory MedicineLos Angeles, California; 7 Institut für Humangenetik, Universität Duisburg-EssenEssen, Germany; 8 Medical Genetics Department, Istanbul Medical Faculty, Istanbul UniversityIstanbul, Turkey; 9 Department of Pediatric Hematology/Oncology, Von Hauner University Children's HospitalMunich, Germany; 10 Sarah M. and Charles E. Seay Centre for Musculoskeletal Research, Texas Scottish Rite Hospital for ChildrenDallas, Texas; 11 Department of Human Genetics, Radboud University Medical CentreNijmegen, The Netherlands; 12 Department of Paediatric Infectious Diseases and Immunology, Radboud University Medical CentreNijmegen, The Netherlands; 13 Nijmegen Institute for Infection, Inflammation and Immunity, Radboud University Medical CentreNijmegen, The Netherlands; 14 Division of Medical Genetics, Department of Pediatrics, University of AlbertaEdmonton, Canada; 15 Department of Clinical Genetics, Discipline of Paediatrics, University of SydneySydney, Australia; 16 The Children's HospitalWestmead, Sydney, Australia; 17 Department of Medical Genetics, Royal North Shore HospitalSt Leonards, NSW, Australia; 18 Sheffield Clinical Genetics Service, Sheffield Children's HospitalSheffield, UK; 19 Sheffield Immunology & Infectious Disease Service, Sheffield Children's HospitalSheffield, UK; 20 Department of Medical Genetics, Cambridge University Hospitals Foundation TrustCambridge, UK; 21 Cambridge Institute of Medical Research, University of CambridgeCambridge, UK; 22 Cologne Center for Genomics (CCG), University of CologneCologne, Germany; 23 Cologne Excellence Cluster on Cellular Stress Responses in Aging-Associated Diseases (CECAD), University of CologneCologne, Germany

**Keywords:** ligase IV, *LIG4*, nonhomologous end joining, radiosensitivity, cytopenia, malignancy, DNA repair, immunodeficiency

## Abstract

Ligase IV syndrome is a rare differential diagnosis for Nijmegen breakage syndrome owing to a shared predisposition to lympho-reticular malignancies, significant microcephaly, and radiation hypersensitivity. Only 16 cases with mutations in *LIG4* have been described to date with phenotypes varying from malignancy in developmentally normal individuals, to severe combined immunodeficiency and early mortality. Here, we report the identification of biallelic truncating *LIG4* mutations in 11 patients with microcephalic primordial dwarfism presenting with restricted prenatal growth and extreme postnatal global growth failure (average OFC −10.1 s.d., height −5.1 s.d.). Subsequently, most patients developed thrombocytopenia and leucopenia later in childhood and many were found to have previously unrecognized immunodeficiency following molecular diagnosis. None have yet developed malignancy, though all patients tested had cellular radiosensitivity. A genotype–phenotype correlation was also noted with position of truncating mutations corresponding to disease severity. This work extends the phenotypic spectrum associated with *LIG4* mutations, establishing that extreme growth retardation with microcephaly is a common presentation of bilallelic truncating mutations. Such growth failure is therefore sufficient to consider a diagnosis of LIG4 deficiency and early recognition of such cases is important as bone marrow failure, immunodeficiency, and sometimes malignancy are long term sequelae of this disorder.

## Introduction

Ligase IV syndrome (MIM #606593) is a disorder of DNA damage repair, with cellular hypersensitivity to ionizing radiation, caused by mutations in *LIG4* (MIM #601837). To date, only 16 cases from 12 families have been described with a high degree of clinical heterogeneity. The first case to be reported was a “developmentally normal” 14-year-old boy (with presumably normal growth) who exhibited severe radiosensitivity during treatment for leukemia [Riballo et al., 1999; Riballo et al., 2001]. *LIG4* mutations were subsequently reported in individuals with microcephaly, mild immunodeficiency, developmental delay, and pancytopenia [O'Driscoll et al., 2001, 2004; Unal et al., 2009]. Ligase IV syndrome was therefore recognized as a rare differential for Nijmegen breakage syndrome (NBS) [Ben-Omran et al., 2005]. Like NBS, where mild short stature is present in many cases [Weemaes, 2000], reduced height has also been reported in some LIG4 cases [Buck et al., 2006b; Gruhn et al., 2007; O'Driscoll et al., 2001; Toita et al., 2007; Unal et al., 2009; Yue et al., 2013]. However unlike NBS, *LIG4* mutations are also described in individuals with severe combined immunodeficiency (SCID) [Buck et al., 2006b; Enders et al., 2006; Grunebaum et al., 2008; van der Burg et al., 2006].

LIG4 is essential for the nonhomologous end joining (NHEJ) of DNA following double strand breaks [Grawunder et al., 1998a]. It complexes with XRCC4, which localizes LIG4 to the site of DNA damage [Berg et al., 2011], where LIG4 acts in the final step of NHEJ, ligating the DNA strands together [Helleday et al., 2007]. Patient cells with *LIG4* mutations have increased sensitivity to DNA double strand breaks induced by ionizing radiation [O'Driscoll et al., 2001]. NHEJ is also required to rejoin the variable, diversity, and joining gene segments at the T-and B-cell receptor loci (V(D)J recombination) [Tonegawa, 1983] as well as in class switch recombination [Casellas et al., 1998; Pan-Hammarstrom et al., 2005]. These processes are essential for adaptive immunity and are defective in LIG4 patients, resulting in SCID in some cases [Buck et al., 2006b; Pan-Hammarstrom et al., 2005; van der Burg et al., 2006]. SCID is also a feature of infants with mutations in three other components of the NHEJ machinery; *DCLRE1C*, encoding Artemis [Moshous et al., 2001], *PRKDC*, encoding the catalytic subunit of DNA-PK (DNA-dependent protein kinase) [van der Burg et al., 2009], and *NHEJ1*, encoding Cernunnos-XLF [Buck et al., 2006a].

Microcephalic primordial dwarfism (MPD) is defined by the presence of both prenatal and postnatal growth restriction resulting in extreme reduction in both head circumference (OFC) and height (more than 4 s.d. below the population mean) [Klingseisen and Jackson, 2011]. MPD represents a group of rare Mendelian disorders, with causative genes affecting cell cycle progression through roles in centriole duplication (*CEP152*, *CENPJ*) [Al-Dosari et al., 2010; Kalay et al., 2011], DNA replication (*ORC1*, *ORC4*, *ORC6*, *CDC6*, *CDT1*, *MCM4*) [Bicknell et al., 2011a; Bicknell et al., 2011b; Gineau et al., 2012; Hughes et al., 2012], ATR-dependent DNA damage response signaling and repair (*PCNT*, *ATR*, *ATRIP*, *RBBP8*) [O'Driscoll et al., 2003; Ogi et al., 2012; Qvist et al., 2011; Rauch et al., 2008] and mitotic spindle organization (*PCNT*) [Zimmerman et al., 2004].

Here, we report the identification of biallelic truncating *LIG4* mutations in a group of patients presenting with MPD. We provide detailed molecular and phenotypic characterization of our patients, and establish that the phenotype in Ligase IV syndrome can be one of severe growth retardation with or without accompanying pancytopenia or SCID.

## Materials and Methods

### Patients

Patients were recruited to research studies at the MRC Human Genetics Unit in Edinburgh, UK, the Institute of Human Genetics at the University of Cologne, Germany and the Nemours Foundation, Delaware by their local clinician. The research studies were approved by the multicenter research ethics committee for Scotland (04:MRE00/19), Cologne hospitals ethics board, and the Nemours Office of Human Subject Protection (NOHSP) & Institutional Review Board respectively. Informed consent was obtained from all families. Medical history, anthropometric data, examination findings, and clinical laboratory results were obtained by questionnaire from the referring physician. All patients selected into the study had a clinical diagnosis of MPD based on head (occipital–frontal) circumference (OFC) and height being more than 4 standard deviations (s.d.) below the population mean at the time of study recruitment (subsequent height measurements in three cases were between −3 and −4 s.d (F4, F6, and F9)). Standard deviations for height, weight, and OFC normalized for age and sex were calculated using Cole's LMS method using UK 1990 cohort data [Freeman et al., 1995]. Age at examination was corrected for prematurity where birth was before 37 weeks gestation and age <2 years. Age was then rounded to the nearest month before s.d. calculation.

### Exome Sequencing and Variant Validation

Genomic DNA was extracted from peripheral blood by standard methods or saliva samples using Oragene collection kits according to manufacturer's instructions. Whole exome capture and sequencing was performed at the Wellcome Trust Sanger Institute (WTSI), UK, and at the University of Cologne, Germany [Asharani et al., 2012]. DNA was sheared to 150 bp lengths by sonification (Covaris, Woburn, MA) before whole exome capture and amplification using the SureSelect Human All Exon 50Mb kit (Agilent, Santa Clara, CA). Fragments were sequenced using Illumina Hiseq platform. 75 bp paired end sequence reads were aligned to the Genome Reference Consortium human build 37 reference sequence using BWA [Li and Durbin, 2009]. Single nucleotide variants were called using GenomeAnalysisTK (http://www.broadinstitute.org/gatk/) and SAMtools (http://www.samtools.sourceforge.net/), whereas Indels were called using Dindel (http://www.sanger.ac.uk/resources/software/dindel/) and SAMtools. Variants were then annotated with functional consequence using Ensembl Variant Effect Predictor [McLaren et al., 2010].

Allele frequency from 1000genomes [Clarke et al., 2012] and dbSNP (http://www.ncbi.nlm.nih.gov/projects/SNP/) databases were used for filtering of population variants. Datasets were analyzed under a model of autosomal recessive inheritance. Variants with the most deleterious effect on protein function (stop gained and frameshift) were prioritized for follow up and confirmed by bidirectional capillary dye-terminator sequencing as previously described [Bicknell et al., 2011b].

Capillary sequencing was performed in the MRC Human Genetics Unit, Edinburgh, UK, (patient F10-sequencing performed in the Erasmus Medical Centre, Rotterdam, The Netherlands). Variants were annotated using the reference sequence, GenBank: NM_002312.3, on the basis of coding sequence with nucleotides numbered from first base of initiation codon (ATG). All variants reported in this article have been submitted to the Leiden Open Variation Database (LOVD v.3.0, http://www.lovd.nl/3.0/home). Primer sequences and PCR conditions for *LIG4* are available on request.

### Haplotype Analysis

Twenty highly polymorphic SNPs (heterozygosity>0.46) present within a 2 Mb region flanking *LIG4* were genotyped by capillary sequencing.

### Statistical Analysis

Percentage of normal hematological parameters was calculated by taking the midpoint of the reference range provided by the patient's diagnostic hematology lab. Significance testing was performed on quantitative data using unpaired and paired Student's *t*-tests.

### Colony Survival Assays

Lymphoblastoid cell lines (LCLs) were derived from peripheral blood lymphocytes and transformed with Epstein–Barr virus. Ionizing radiation clonogenic survival assays were performed using a previously validated method [Sun et al., 2002] in which dose–response curves were initially generated to identify suitable doses of ionizing radiation needed to discriminate wild-type and radiosensitive LCLs.

## Results

### Compound Heterozygous Truncating Mutations in *LIG4* Cause Extreme Growth Failure

Exome sequencing performed in 55 patients selected from a cohort of 138 patients with MPD, identified *LIG4* mutations in five patients. Initially, with variant filtering performed removing all dbSNP variants with assigned “rs” accession numbers, only one heterozygous truncating mutation in *LIG4* was apparent in each patient. However, refiltering the dataset to include all variants with an allele frequency of less than 0.01 in the 1000 genomes dataset [Clarke et al., 2012], identified a second mutation, c.2440C>T (p.Arg814*, rs104894419, allele frequency 0.003), in all five cases.

Mutations were subsequently identified in six additional individuals, by capillary sequencing *LIG4* in the remaining patients from the same MPD cohort who had not been exome sequenced. A further patient was identified with a single heterozygous truncating mutation, c.128delT (p.Leu43*), however, a second pathogenic variant was not identified. Therefore, in total, 11 patients from 10 families were identified with biallelic truncating mutations in *LIG4* from 138 MPD patients (Table 1).

**Table 1:** *LIG4* Mutations Identified in 10 Families (F1–10) with a Clinical Diagnosis of Microcephalic Primordial Dwarfism

Patient	Nucleotide change	Protein change	Country of Origin	Mother	Father	Size of p.Arg814^*^ associated haplotype
F1.1	c.[2440C>T]	p.(Arg814^*^)	Canada	p.(Arg814^*^)	p.(Lys424Argfs^*^20)	1.49 Mb
	+[1271_1275delAAAGA]	p.(Lys424Argfs^*^20)				
F1.2	c.[2440C>T]	p.(Arg814^*^)	Canada	p.(Arg814^*^)	p.(Lys424Argfs^*^20)	1.49 Mb
	+[1271_1275delAAAGA]	p.(Lys424Argfs^*^20)				
F2	c.[2440C>T]	p.(Arg814^*^)	USA	p.(Arg814^*^)	N/A	1.29 Mb
	+[2094C>G]	p.(Tyr698^*^)				
F3	c.[2440C>T]	p.(Arg814^*^)	Australia	p.(Arg814^*^)	p.(Ala797Aspfs^*^3)	None
	+[2386_2389dupATTG]	p.(Ala797Aspfs^*^3)				
F4	c.[2440C>T]	p.(Arg814^*^)	UK	p.(Lys424Argfs^*^20)	p.(Arg814^*^)	>2 Mb
	+[1271_1275delAAAGA]	p.(Lys424Argfs^*^20)				
F5	c.[2440C>T]	p.(Arg814^*^)	USA	p.(Arg814^*^)	p.(Lys424Argfs^*^20)	None
	+[1271_1275delAAAGA]	p.(Lys424Argfs^*^20)				
F6	c.[2440C>T]	p.(Arg814^*^)	Germany	p.(Arg814^*^)	p.(Lys424Argfs^*^20)	1.19 Mb
	+[1271_1275delAAAGA]	p.(Lys424Argfs^*^20)				
F7	c.[2440C>T]	p.(Arg814^*^)	USA	N/A	N/A	N/A
	+[1512_1513delTC]	p.(Arg505Cysfs^*^12)				
F8	c.[2440C>T]	p.(Arg814^*^)	UK	N/A	N/A	>2 Mb
	+[1246_1250dupGATGC]	p.(Leu418Metfs^*^3)				
F9	c.[2440C>T]	p.(Arg814^*^)	Turkey	p.(Arg814^*^)	p.(Lys424Argfs^*^20)	None
	+[1271_1275delAAAGA]	p.(Lys424Argfs^*^20)				
F10	c.[613delT]	p.(Ser205Leufs^*^29)	The Netherlands	p.(Ser205Leufs^*^29)	p.(Lys635Argfs^*^10)	N/A
	+[1904delA]	p.(Lys635Argfs^*^10)				

F1.1 and F1.2 represent individual affected siblings. All mutations are predicted to cause premature truncation of the protein. Nucleotides numbered from first base of initiation codon (ATG) in coding DNA sequence [GenBank: NM_002312.3]. N/A, not available.

Biallelic *LIG4* mutations were therefore present in 9% of MPD cases screened without a previous molecular diagnosis and represented 2.4% of our total MPD families. Forty-three cases with primary microcephaly and 56 with microcephaly and milder short stature (height between −2 and −4 s.d. from the population mean) were also sequenced but no further mutations were identified in these patient groups.

Where parental DNA was available, all parents were found to be healthy heterozygous carriers. Eight different truncating mutations were identified in total, six of which had not been previously described. Nine out of 11 affected patients were females, and likewise the majority of published cases are also female (nine female, two male, three unspecified). However, the reason for such potential skewing in sex ratio is not readily evident.

c.2440C>T (p.Arg814*) was identified recurrently, present in 10 of the 11 cases. High density genotyping of SNPs surrounding *LIG4* in 10 families harboring c.2440C>T identified a common haplotype of at least 2 Mb in the two families, F4 and F8, establishing that c.2440C>T represented a founder mutation (Supp. Fig. S1). Three further families, F1, F2, and F6, also shared 6–12 SNPs of the same haplotype located adjacent to the gene suggesting the mutation could share the same ancestral origin in these patients too. Importantly, the mutation was also observed in other haplotypes in three of the patients (F3, F5, F9), establishing that c.2440C>T is a disease-causing mutation, independent, and distinct from other co-segregating variants. c.2440C>T coincides with a CpG site and could therefore represent a mutational hotspot. c.1271_1275delAAAGA (p. Lys424Argfs*20) was present in six families however no common haplotype associated with this mutation was observed.

### Truncating *LIG4* Mutations Cause Severe Pre-and Postnatal Growth Failure

**Figure 1:**
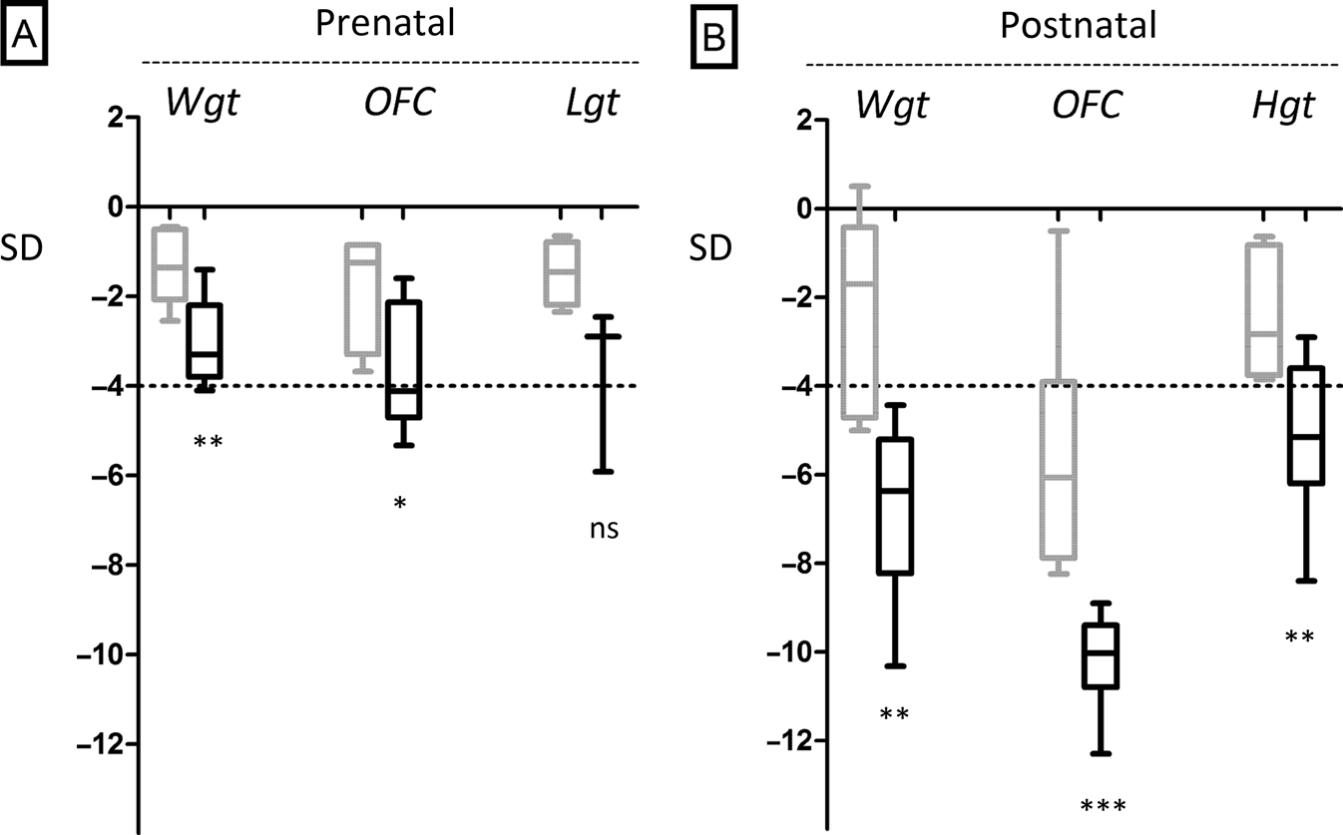
Growth is significantly more impaired both at birth and postnatally in 11 cases with *LIG4* mutations in comparison with previously described patients. Measurements plotted as *Z*-scores (standard deviation of measurement from population mean for age and sex) for previously reported cases (gray) alongside the patients reported here (black) for each growth parameter. A: Measurements at birth. B: Most recent postnatal measurements. Box indicates 95% confidence interval with horizontal bars at mean. Range indicated by vertical bars. Average measurements in previously reported cases where morphometric data were provided [Ben-Omran et al., 2005; Buck et al., 2006b; Gruhn et al., 2007; Grunebaum et al., 2008; Toita et al., 2007; van der Burg et al., 2006; Yue et al., 2013] were: mean birth weight −1.4 ± 0.8 s.d. (*n* = 7), birth OFC −1.9 ± 1 s.d. (*n* = 5) and birth length −1.5 ± 0.7 s.d. (*n* = 4), postnatal height −2.4 ± 1.5 s.d. (*n* = 5); weight −2.4 ± 2.3 s.d. (*n* = 5); and OFC −5.6 ± 2.8 s.d. (*n* = 6). Abbreviations: OFC, occiptofrontal circumference; Wgt, weight; Lgt, length; Hgt, height; ns, not significant. *P < 0.05; ^**^P < 0.01; ^***^P < 0.001.

Intrauterine growth retardation (IUGR) was present in all cases; mean birth weight −3.0 ± 0.93 s.d. (*n* = 11), birth OFC −3.6 ± 1.37 s.d. (*n* = 8), and length −3.8 ± 1.88 s.d. (*n* = 3) (Fig. 1). Postnatal weight, OFC, and length were also significantly reduced in all cases; weight −6.8 ± 1.96 s.d., OFC −10.1 ± 0.95 s.d., and length −5.1 ± 1.62 s.d. Therefore, disproportionate microcephaly was present, with postnatal head circumference substantially more reduced than height (*P* = 0.0001). Growth failure in the current series of patients resulted in extreme postnatal short stature and microcephaly that was significantly greater than previously reported cases (Fig. 1).

### Facial Features, Malformations, and Cognition in *LIG4* Mutation Positive Patients

**Figure 2:**
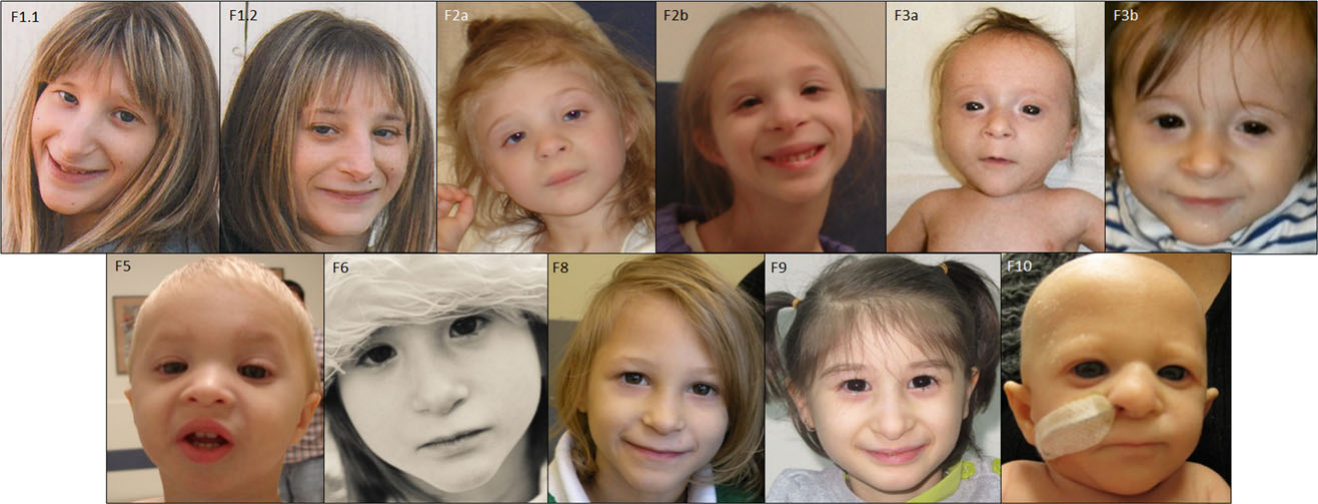
Facial features of six LIG4 cases with microcephalic primordial dwarfism. Age of patient at time of photos; F1.1 20 years, F1.2 14 years, F2 (a) 3 years and (b) 6 years 10 months, F3 (a) 3 months and (b) 10 months, F5 20 months, F6 4 years, F8 6 years, F9 6yrs 2 months, and F10 5 months.

All patients had a similar facial appearance (Fig. 2). Common features in early childhood included fine sparse hair, epicanthic folds, wide depressed nasal bridge, broad nasal tip, and a prominent chin. No hypodontia was reported (a characteristic feature of patients with mutations in *PCNT* (MOPDII) [Rauch et al., 2008]). A number of malformations were present (Table 2), although these were individually at low frequency, as observed in NBS [Weemaes, 2000]. Congenital hip dysplasia was the only commonly reported abnormality, occurring in three individuals (F3, F5, F6). Severe vomiting episodes were also reported in one case (F6), the cause of which has not yet been determined. These episodes have occurred from birth and resulted in significant feeding difficulties throughout childhood. Primary ovarian failure was also reported in both female patients who have so far reached pubertal age.

**Table 2:** Anthropometric Data and Clinical Findings

			A. Anthropometric Data					B. Clinical Data	
Pt	Sex	Gest^n^ /weeks	BW/s.d. (kg)	Birth OFC /s.d. (cm)	Age at examination	OFC/s.d. (cm)	Height/s.d. (cm)	Developmental Delay	Malformations and additional features
F1.1	F	38	−3.6	−4.87	17y6m	−9.4	−6	Mild	Small cerebral aneurysm, primary ovarian failure
			(1.59)	(27.75)		(42.5)	(127)		
F1.2	F	40	−2.2	N/A	11y9m	−9.9	−4.3	Mild–moderate	Atrial–ventricular septal defect, atrophic kidney, rib hypoplasia, fusion of carpal bones, copper beaten skull, platybasia, abnormal C1 vertebrae and primary ovarian failure
			(1.96)			(41.5)	(117.5)		
F2	F	33	−3.3	−4.07	7y10m	−12.3	−5	Mild	Anal atresia with rectovaginal fistula, esotropia
			(1.01)	(25)		(37.9)	(99)		
F3	F	37	−3.3	−4.17	2y2m	−10.8	−6.3	Mild	Unilateral congenital hip dysplasia, cutis marmorata
			(1.58)	(28)		(36.3)	(68.5)		
F4	F	40	−3.8	N/A	2y6m	−8.9	−3.6	None	Psoriasis
			(1.84)			(39)	(78.4)		
F5	M	38	−1.6	−2.72	2y	−9.1	−5.3	None	Unilateral congenital hip dysplasia
			(2.44)	(30.5)		(38.2)	(70.4)		
F6	F	32	−2.95	−1.94	2y	−10.21	−3.08	Mild	Congenital hip dysplasia, 2/3 toe syndactyly, excessive vomiting (gastrostomy in situ)
			(0.95)	(27)		(36.5)	(75)		
F7	F	37	−4.1	−4.21	3y8m	−10.03	−5.15	None	None
			(1.29)	(27.5)		(38.8)	(79)		
F8	F	34	−2.93	N/A	1y9m	−9.6	−6.2	Mild	None
			(1.23)			(37)	(65)		
F9	M	37+	−1.4	−1.6	5y6m	−10.8	−2.9	Mild	Hypopigmentation, hypermobile knees, single palmar crease, 2/3 toe syndactyly, sandal gap
			(2.25)	(30.8)		(39)	(98)		
F10	M	37	−4.0	−5.33	3m	−10.1	−8.4	N/A	Pre-axial polydactyly 2/5 toe syndactyly, dysplastic kidney and dysgenesis of corpus callosum.
			(1.3)	(26.5)		(29)	(43)		

Anthropometric data stated as *Z* scores (standard deviation from population mean for age and sex), actual measurements in brackets.

Gest^*n*^, gestation; BW, birth weight; OFC, occipitofrontal circumference; s.d., standard deviation; N/A, not available; m, month; y, year.

Development was reported to be normal in three cases, mildly delayed in six and moderately delayed in one (Table 2). In those in which developmental delay had been reported, impairment in expressive language skills was predominant. All school-aged children were in mainstream school with additional support being provided where required. One patient died at the age of 6 months following severe infections from birth and therefore developmental data is not available (F10).

### Marked Thrombocytopenia with Milder Leukopenia Occurs Frequently in Early to Mid-Childhood

**Figure 3:**
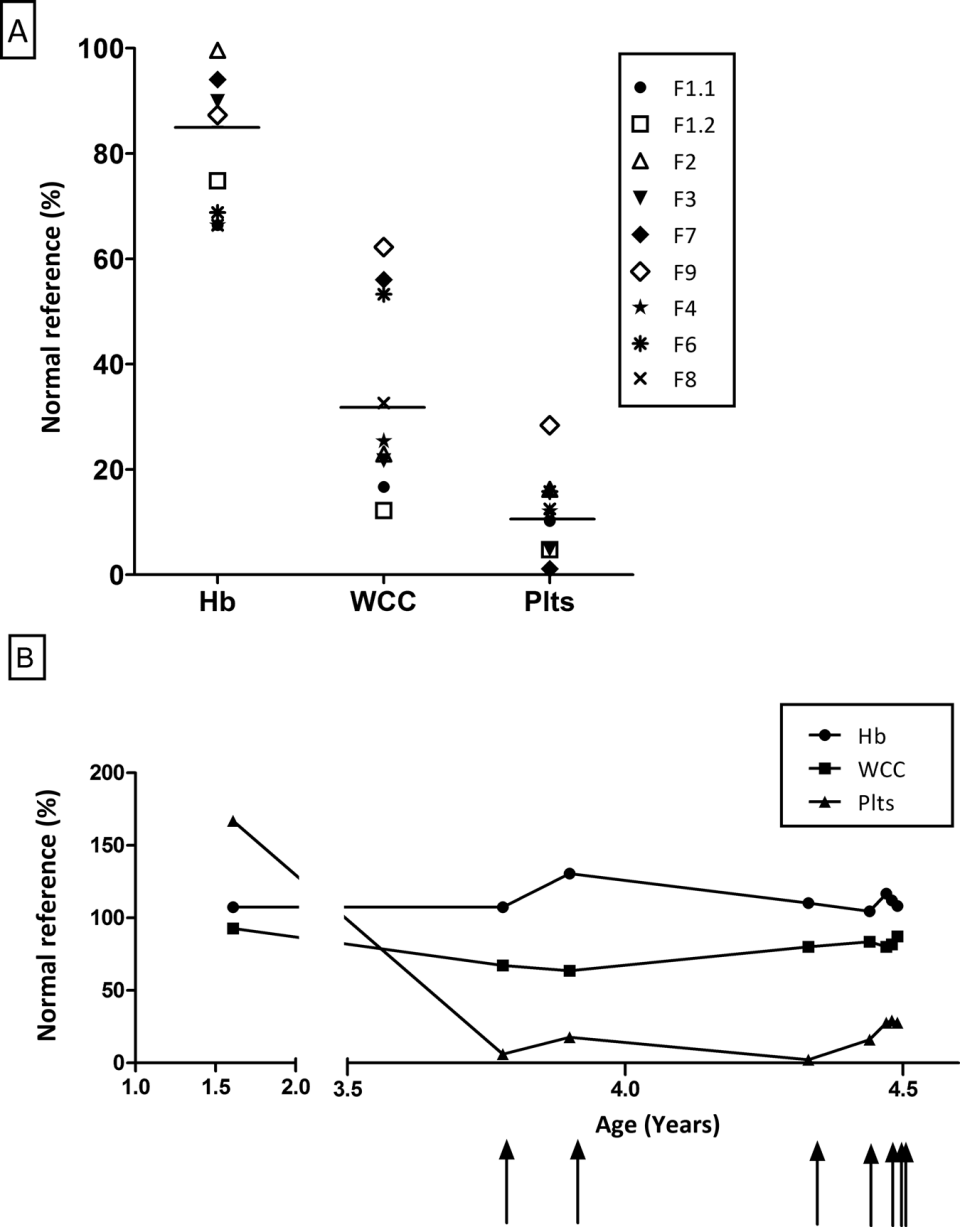
Platelets are the most significantly affected cell type in cytopenic episodes in patients with *LIG4* mutations and severity of episodes increases with age. Values are shown as percentage of average normal count. A: Documented blood counts from nine patients during the most recent cytopenic episode. B: Serial blood counts in one patient (F8) over time demonstrating increasing frequency of transfusions with age. Arrows indicate when platelet transfusion occurred.

Nine patients (>80%) have had documented cytopenia requiring platelet transfusions with no apparent trigger (Table 2). In seven of these patients, the first episode occurred after recruitment to the study (Table 3), with the earliest episode at 2 years of age (F3 and F4) and the latest at 15 years (F1.1). In all the cases, platelets were the most significantly affected cell type (calculated as percent of normal; 11.8% ± 8 s.d., *P* = 0.0001) followed by leukocytes (33.7% ± 19 s.d., *P* = 0.0001). Hemoglobin levels were only mildly reduced (79.3% ± 13 s.d., *P* = 0.002) (Fig. 3A). Bone marrow aspiration performed in four individuals demonstrated a hypocellular marrow without morphological abnormalities. In eight patients, a progressive course of bone marrow failure was evident with increasing transfusion frequency with age (Fig. 3B). However, Patient F1.1 is currently no longer transfusion dependent although indices remain subnormal on recent investigation (Table 3). Lympho-reticular malignancy has not been observed in any of the 11 cases with the oldest patient being 21years at time of publication.

**Table 3:** Clinical Course of Patients with Further Details of Hematological and Immune Investigation

				B. Current Hematological Test Results							C. Current Immunological Test Results					
A. Features at referral							WCC					Immunoglobulins g/dl				
*Pt*	*Age of referral to study*	*Hematology*	*Increase in Infection*	*Onset of cytopenia*	*Age at measurement shown*	*Hb g/dl*	*Total 10^3^/μl*	*Neut %*	*Lymph %*	*Plts 10^3^/μl*	*Age at measurements shown*	*IgG*	*IgA*	*IgM*	*Lymphocyte subset Cells/μl*	*Antibody response to Vaccination*
F1.1	17y	6month period pancytopenia – self resolved	Mild	15y	20y	**8.2**	**1.5**	53	40	**30**	15y	**4.31**	1.06	0.14	CD3 680, CD8 380, **CD4 240, CD19 0**	N/A
															Naïve CD4 and CD8 counts very low	
F1.2	14y	Persistant pancytopenia	Mild	8y	14y	**10.1**	**1.1**	36	64	**14**	14y	**5.59**	1.29	0.76	CD3 660, CD8 370, **CD4 190, CD19 10**	N/A
															Naïve CD4 and CD8 counts very low	
F2	3y	One cytopenic episode self-resolved	None	3y	3y	12.6	**1.8**	55	24	**43**	In progress	N/A	N/A	N/A	N/A	N/A
F3	4m	None	Mild	2y	2y	11.1	**2**	30	40	**18**	2y	**3.7**	0.58	0.74	**CD3 710, CD8 170**, CD4 500, **CD19 very low**	Poor response to pneumococcal vaccine
F4	2y	None	None	2y	6y	**8.2**	**2.34**	N/A	N/A	**32**	6y	**Low**	N/A	N/A	**CD4 low and CD19 very low**	Poor response to pneumococcal vaccine
F5	1y	None	None	Not apparent at 3y	N/A	N/A	N/A	N/A	N/A	N/A	2y	**3.66**	0.8	0.62	**CD3 467, CD8 204, CD4 164, CD19 6Total lymph count reduced**	N/A
F6	Neonate	None	Mild	3y 10m	3y 10m	**8.5**	**4.9**	55	32	**42**	4y	**5.76**	4.23	0.27	CD3 983, CD8 292, CD4 583, **CD19 11**	Tetanus and diphtheria normal, HiB and pneumococci low
F7	3y8m	None	None	3y9m	4y4m	11.9	**4.9**	54.3	26	**3**	In progress	N/A	N/A	N/A	N/A	N/A
F8	4y	None	None	6y	6y2m	**8.2**	**3**	N/A	N/A	**33**	6y	**4.8**	2.4	<0.3	N/A	N/A
F9	2y6m	None	Mild	6y	6y	11.7	5.6	60.8	27.2	**78.1**	5y6m	9.7	3.1	1.0	CD3, CD8 & CD4 normal, **CD19 very low**	N/A

Increase in infections: “Mild” defined as an increased frequency of common pathogens (upper and lower respiratory, skin, and gastrointestinal) or increased severity of a single illness above that deemed normal for childhood by their clinician. Absolute full blood count values are the most recent recorded in which cytopenia is demonstrated. Normal range of absolute counts varies with age, sex, and laboratory methods but approximate reference ranges are as follows: Hb 10.8–13.9 g/dl, Total WCC 5.5–12.9×10^3^/μl, Neut 30%–60%, Lymph 20%–50%, Plts 130–400 ×10^3^/μl, IgG 5.65–17.65 g/dl, IgA 0.85–3.85 g/dl, IgM 0.55–3.75 g/dl, Total T lymphocytes (CD3+) 870–2080/μl, T helper subset (CD4+) 530–1290/μl, T cytotoxic subset (CD8+) 220–960/μl, B lymphocytes (CD19+) 100–400/μl. Levels considered below normal range (according to local laboratory) are highlighted in bold. Results for patient F10 are detailed in Ijspeert et al. (2013).

Hb, hemaglobin; WCC, white cell count; Plts, platelet count; Neut, Neutrophil count; Lymph, lymphocyte count; BMT, bone marrow transplant; N/A, not available or not performed; m, month; y, year.

### Impaired Adaptive Immunity is Apparent on Immunological Investigation

At recruitment to the study, immunodeficiency was not clinically suspected in 10 of the 11 cases. Retrospectively, several children (F1.2, F3, F6, F9) have had recurrent common childhood illnesses (respiratory, skin, and gastrointestinal) and one patient (F1.1) had a severe influenza illness requiring hospitalization despite vaccination. In one patient (F10), a diagnosis of SCID became clinically apparent at an early age with recurrent infections from birth and death at 6 months because of an acute gastric bleed in association with a clinical sepsis syndrome. Immune function was noted to be profoundly impaired on investigation in this patient [Ijspeert et al., 2013].

Subsequent to diagnosis with Ligase IV deficiency, detailed immunological investigations have been performed in many of the remaining patients (Table 3), demonstrating hypogammaglobulinemia in seven of the eight patients tested, and in the seven patients for which T/B cell subset data are available, B cell counts were severely depleted, alongside less marked T cell subset deficiencies. Bone marrow transplantation (BMT) is now underway in patient F3, and is being considered in several others (F1.2, F6, and F7).

### Repair of IR Induced DSB is Impaired in Patient Cells

Radiosensitivity of LIG4 lymphoblastoid cells was examined using a colony survival assay previously established for the testing of A-T patients [Sun et al., 2002]. Increased sensitivity to DNA damage was seen in all three patients tested (F2, F5, and F7), with reduced survival of cells (3%–5% cell survival) following exposure to 1 Gray of ionizing radiation (lab reference range for wild-type cells: 50.1% ± 13.5, *n* = 24). This is similar to the sensitivity seen in ataxic telangectasia patient cells (13.1% ± 7.2, *n* = 104) and that observed in previous studies of LIG4 patients [O'Driscoll et al., 2001]. Increased cellular sensitivity to ionizing radiation is also present in patient F10 [Ijspeert et al., 2013].

### Phenotype Severity Correlates with the Position of the Truncating Mutation

**Figure 4:**
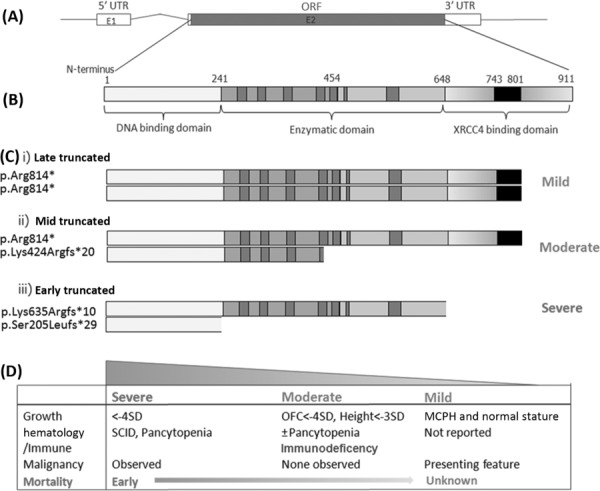
Degree of protein truncation correlates with severity of growth failure and immuno deficiency. A: Schematic of the *LIG4* gene, which contains two exons, with exon 2 encoding the 911aa protein. B: LIG4 protein domain structure: The protein is composed of an enzymatic domain in which highly conserved sequence motifs have been identified (dark gray) [Marchetti et al., 2006; Shuman and Schwer, 1995], and a C-terminal XRCC4 binding domain composed of 2 BRCT domains (light gray) with a linker region that contains the minimal XRCC4 binding motif (black) [Sibanda et al., 2001]. C and D: Position of truncating mutations correlating with phenotype. D: Biallelic mutations representative for each group are depicted: (i) Late truncating; a homozygous c.2440C>T, p.Arg814* mutation, represents the mildest truncating mutation reported, only affecting the latter part of the XRCC4 binding domain and is associated with normal development and a mild growth phenotype [Ben-Omran et al., 2005]. (ii) Late and mid truncating; the majority of cases described in this manuscript (F1.1-F9) carry the c.2440C>T mutation combined with a more proximally truncating mutation associated with a severe growth phenotype, chronic or progressive cytopenia and immune dysfunction. (iii) Mid and early truncating mutations; the most severely affected case with SCID (F10) carried a “mid-truncating” mutation which completely removed the XRCC4 binding site in combination with an “early” truncating mutation that removed the entire enzymatic domain. Abbreviations: ORF, open reading frame; UTR, untranslated region.

The coding sequence for *LIG4* is solely in the terminal exon (Fig. 4) and therefore transcripts should not be targeted for nonsense mediated decay. All mutations identified in our cohort were predicted to truncate the LIG4 protein to varying degrees, and position of truncating mutations correlated with the phenotype severity observed (Fig. 4).

## Discussion

### The Phenotype Spectrum of Patients with *LIG4* Mutations

Here, we report the identification of *LIG4* mutations in 11 patients with profound global growth failure, expanding the phenotypic spectrum associated with dysfunction of the ligase IV enzyme. Previously, mutations have been associated with clinical presentations ranging from lympho-reticular malignancy in childhood, to severe immunodeficiency (SCID) with mortality in the first few months of life [Ben-Omran et al., 2005; Buck et al., 2006b; Enders et al., 2006; Grunebaum et al., 2008; O'Driscoll et al., 2001; O'Driscoll et al., 2004; Riballo et al., 1999; Riballo et al., 2001; Toita et al., 2007; Unal et al., 2009; van der Burg et al., 2006]. Microcephaly is common to both previously reported and current cases occurring in more than 80% of all reported patients to date. Although reduced stature has been noted in some cases [Buck et al., 2006b; Gruhn et al., 2007; Grunebaum et al., 2008; O'Driscoll et al., 2001; Toita et al., 2007; Unal et al., 2009; Yue et al., 2013], such severe global growth failure has not been previously documented. Given the substantial number of additional LIG4 patients identified by our study, the “MPD” phenotype represents a previously under recognized group of LIG4 patients. However, this phenotype might well have been anticipated given the ∼40% reduction in body size reported in two *LIG4* hypomorphic mouse models [Nijnik et al., 2007; Rucci et al., 2010].

The marked growth phenotype in our cohort might suggest that these cases could be molecularly distinct from previous reports. In contrast to previous cases with primarily missense mutations, all of our patients had compound heterozygous truncating *LIG4* mutations. As all these truncating mutations are in the terminal exon (and therefore would not be expected to be susceptible to nonsense mediated decay), transcripts encoding truncated proteins are predicted to be expressed at normal levels. Notably, disease severity correlated with position of mutation (Fig. 4), with two “early” truncating mutations having the most severe phenotype with SCID, while the combination of an “early” truncating mutation with the c.2440C>T (p.Arg814*) mutation led to global growth reduction without clinically apparent immunodeficiency before investigation. Three cases with a similar combination of truncating mutations have been reported previously. Anthropometric data are unavailable for two of these patients [O'Driscoll et al., 2001], however, a third recently identified patient has a similar clinical picture to our patient group [Yue et al., 2013]. Additionally, a previously reported patient, with c.2440C>T /c.2440C>T mutations had a milder phenotype, with microcephaly but normal stature [Ben-Omran et al., 2005] and without clinical suspicion of immunodeficiency or cytopenia. Therefore, the degree of protein truncation appears to correlate with phenotype severity.

Previous biochemical and cellular studies may explain this correlation, as the c.2440C>T mutation impairs enzymatic function less than earlier truncating mutations [Girard et al., 2004]. Codon Arg814 lies at the start of the second BRCT domain of the XRCC4 binding site [Critchlow et al., 1997]. Loss of this BRCT domain impairs XRCC4 binding [Girard et al., 2004], although the major determinant of XRCC4 binding, the “intervening linker sequence,” is retained [Grawunder et al., 1998b]. As XRCC4 binding is required for LIG4 stability [Bryans et al., 1999], the p.Arg814* mutation results in impaired LIG4 cellular activity, with reduced LIG4 protein levels [O'Driscoll et al., 2001] and also impairs ligase activity [Girard et al., 2004]. Earlier (close to the N-terminus) protein truncation leads to a more complete loss of function, with truncated protein resulting from the c.2094C>G (p.Arg580*) mutation not interacting with XRCC4 or localizing to the nucleus [Girard et al., 2004].

### Clinical Implications: Diagnosis and Management

The availability of clinical data from a series of 11 patients permits a more comprehensive description of common facial, developmental, and hematological features for Ligase IV syndrome, which could be diagnostically useful. A facial gestalt is apparent across the patient group, with fine sparse hair, epicanthic folds, wide depressed nasal bridge, broad nasal tip, and prominent chin in early childhood (Fig. 2), in keeping with the facial features of a previously reported case [Gruhn et al., 2007]. Notably these features are somewhat reminiscent of those described in Dubowitz syndrome, in which severe microcephaly and IUGR also occurs [Tsukahara and Opitz, 1996] and had been previously considered as a differential in some of our patients. Furthermore, a patient initially diagnosed with Dubowitz syndrome has recently been reported with biallelic truncating mutations in *LIG4* [Yue et al., 2013].

Bone marrow failure appears to be a strongly discriminative diagnostic feature of Ligase IV syndrome, occurring in ∼70% of all reported cases to date. However, cytopenia may not be evident at initial presentation, particularly in very young children. *LIG4* mutations should also be considered in the differential diagnosis of Fanconi anemia syndrome, especially in patients with microcephaly and growth failure but without limb reduction defects. In contrast to many previous reports [Buck et al., 2006b; Enders et al., 2006; Grunebaum et al., 2008; van der Burg et al., 2006], immunodeficiency was not suspected clinically before molecular diagnosis in the majority of patients but further investigation has revealed significant humoral and cellular immunodeficiency. This highlights the need for early recognition of Ligase IV deficiency and subsequent immunological investigation even in asymptomatic patients.

Predisposition to malignancies is common in disorders with defective double-strand DNA break damage repair such as ataxia-telangiectasia and NBS [Nahas and Gatti, 2009]. Although no cases of malignancy have occurred in our case series, malignancies have been described in 25% of LIG4 cases overall. Although predominantly lympho-reticular [Ben-Omran et al., 2005; Buck et al., 2006b; Enders et al., 2006; Riballo et al., 1999; Toita et al., 2007], a case of anal carcinoma at the age of 34 years has recently been reported [Yue et al., 2013]. As our patients’ cells exhibit sensitivity to ionizing radiation comparable to that reported previously [O'Driscoll et al., 2001], an increased malignancy risk should be anticipated [Nahas and Gatti, 2009]. Avoidance of nonessential exposure to ionizing radiation is clearly desirable in LIG4 patients. Likewise, if BMT becomes necessary, modification of conditioning regimes appears to be important. Additionally, increased sensitivity to the immunosuppressant, Cyclosporin A has also been suggested [O'Driscoll and Jeggo, 2008] and successful transplantation has now been reported [Enders et al., 2006; Gruhn et al., 2007; Grunebaum et al., 2008; Unal et al., 2009].

Given the hypersensitivity to radiation, Ligase IV syndrome is an important diagnosis to make [Nahas and Gatti, 2009]. It is a relatively frequent cause of MPD, a phenotype group with high genetic heterogeneity. Only mutations in *PCNT* (20%) and *RNU4atac* (2.4%) are seen as frequently in our experience (unpublished data, LSB, JEM, APJ). c.2440C>T (p.Arg814*) has been previously identified in European populations, with an allele frequency of 0.001 and 0.003, in Exome Variant Server and “1000 Genome” databases, respectively. Therefore, there may be many unrecognized cases of LIG4 deficiency. In particular, the frequency of c.2440C>T homozygotes would be expected to be 1 in 100,000 to 1 in 1,000,000, potentially presenting as nonsyndromic microcephaly with normal development [Ben-Omran et al., 2005]. For those that reach the genetics clinic, the routine adoption of high-throughput sequencing in molecular diagnostics is likely to improve ascertainment; however, appropriate setting of allele frequency filters is crucial. This is well illustrated by our initial oversight of the *LIG4* mutations when all dbSNP annotated variants, including the c.2440C>T mutation, were removed.

In summary, we report that mutations in *LIG4* occur in patients presenting with extreme growth failure, extending the phenotypic spectrum of Ligase IV deficiency. We establish that a recurrent mutation present in conjunction with a more severely truncating mutation confers profound growth impairment with progressive bone marrow failure and immunodeficiency evident on laboratory investigation.
