# Supplementary figures and images for: Extreme Growth Failure is a Common Presentation of Ligase IV Deficiency
Source: Hum Mutat. 2013 Nov 8;35(1):76–85. doi: 10.1002/humu.22461 (PMC3995017; doi:10.1002/humu.22461)

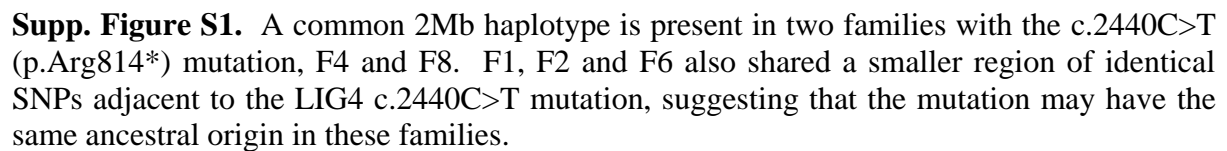

Supplement: Supplementary file 1 [file humu0035-0076-sd1.pdf]
